# Experimental Design and Sample Preparation in Forest Tree Metabolomics

**DOI:** 10.3390/metabo9120285

**Published:** 2019-11-22

**Authors:** Ana M. Rodrigues, Ana I. Ribeiro-Barros, Carla António

**Affiliations:** 1 Plant Metabolomics Laboratory, Instituto de Tecnologia Química e Biológica António Xavier, Universidade Nova de Lisboa (ITQB NOVA), 2780-157 Oeiras, Portugal; amrodrigues@itqb.unl.pt (A.M.R.); aribeiro@isa.ulisboa.pt (A.I.R.-B.); 2 Plant Stress and Biodiversity Laboratory, Linking Landscape, Environment, Agriculture and Food (LEAF), Instituto Superior de Agronomia, Universidade de Lisboa (ISA/ULisboa), 1349-017 Lisboa, Portugal

**Keywords:** plant metabolomics, forestry, trees, mass spectrometry, metabolite extraction, GC-MS, LC-MS, metadata standardization, databases

## Abstract

Appropriate experimental design and sample preparation are key steps in metabolomics experiments, highly influencing the biological interpretation of the results. The sample preparation workflow for plant metabolomics studies includes several steps before metabolite extraction and analysis. These include the optimization of laboratory procedures, which should be optimized for different plants and tissues. This is particularly the case for trees, whose tissues are complex matrices to work with due to the presence of several interferents, such as oleoresins, cellulose. A good experimental design, tree tissue harvest conditions, and sample preparation are crucial to ensure consistency and reproducibility of the metadata among datasets. In this review, we discuss the main challenges when setting up a forest tree metabolomics experiment for mass spectrometry (MS)-based analysis covering all technical aspects from the biological question formulation and experimental design to sample processing and metabolite extraction and data acquisition. We also highlight the importance of forest tree metadata standardization in metabolomics studies.

## 1. Introduction

Metabolomics is an “omics” technology used to obtain comprehensive information on the metabolome: a diverse pool of low molecular weight molecules (metabolites), present in a cell or organism, and at a particular physiological or developmental stage [1]. For the past 20 years, the number of mass spectrometry (MS)-based metabolomics studies in plants has grown exponentially and plant metabolomics has established itself as a powerful tool to address biological questions related to plant growth and development and plant responses to environmental perturbations [2,3]. Despite the continuous advances in MS technology, the coverage of the plant metabolome is a major challenge in plant metabolomics research mainly due to the high chemical diversity, broad dynamic range of concentration, and specific cellular compartmentalization of metabolites. In addition, no single analytical technology can cover the entire plant metabolome, and different extraction techniques and combinations of complementary analytical technologies are often employed [4]. In general, preparing a plant sample for a metabolomics study involves the establishment of a good experimental design, followed by several standard steps for sample preparation, namely: harvest immediately followed by quenching, aliquot weighing, metabolite extraction, pre-analytical procedures (if required, e.g., chemical derivatization), and finally, metabolite analysis [2,5,6,7]. The standardization of these metabolomics workflows ensures data consistency and allows the reproducibility of the generated data and metadata (information about data origins). Although most steps are common to any metabolomics experiment, the optimization of laboratory procedures is often adopted, according to the requirement of the sample (species or tissue) under study. This is particularly the case of metabolomics studies on tree species. Forest tree metabolomics represents additional challenges when compared to other plant metabolomics studies. These include an experimental design that takes into account the long life cycle and the genetic variability of forest tree species as well the presence of interferents that can require additional steps during sample preparation (e.g., additional concentration steps) [8]. In this review, we highlight the major challenges when setting up an MS-based forest tree metabolomics experiment. Although this review is focused mainly on forest tree species, the methodology here reviewed can be applied to other woody species.

## 2. Experimental Design for Forest Tree Metabolomics

In a plant metabolomics study, after the formulation of the biological question, experimental design planning is the first crucial step of the metabolomics workflow. The experimental design includes the complete planning of the experiment, including plant growth conditions and the treatments to be applied to the plants. In this section, all the critical steps and important decisions for a good experimental design are discussed.

### 2.1. Biological Question Formulation

A plant metabolomics experiment starts with the formulation of a good hypothesis (i.e., biological question) to plan an appropriate experimental design, sample preparation, and statistical strategies for data analysis. Without a clear biological question, the observed changes can be misinterpreted or have multiple possible interpretations that would not reveal important information related to the biological system. Thus, it is absolutely crucial to understand the biological system under study to not only select the suitable tissue(s) for analysis but also the appropriate controls. Understanding the biological system will allow the elaboration of an accurate experimental design, and ultimately, to answer the biological question. It is important to highlight that frequently (and wrongly) experiments are designed for other “omics” technologies (i.e., transcriptomics, proteomics), and the leftover samples are later used for metabolomics analysis. This can extensively compromise the entire metabolomics analysis because the objective of the study might be different, the number of replicates may not be sufficient, or the sample storage conditions were not ideal, thereby affecting the stability of metabolites within the sample [9].

Forests represent a crucial driver to achieve the sustainable development goals (SDG) from Agenda 2030 of the United Nations through the provision of a wide range of ecosystem goods and services with a direct impact on socio-economic development and environmental balance [10,11]. In addition to the direct economic benefits provided by tree species, i.e., timber and non-timber products, gaming and tourism, forests have an immensurable ecological value, being the major determinants for water, oxygen, carbon, and energy balance and can be seen as a major opportunity to mitigate climate change effects [12], i.e., continued drought, increased soil and water salinization and acidification, and intensification of extreme temperatures [13]. In forest tree metabolomics research, most biological questions are indeed related to the responses towards the acclimation and adaptation to a permanently changing environment [14,15,16,17,18,19,20,21,22,23,24,25,26] as well as to the identification of potentially active components in tree species of pharmacological, agricultural, environmental, or industrial importance [27,28,29,30,31,32,33].

### 2.2. Experimental Design

**Figure 1:**
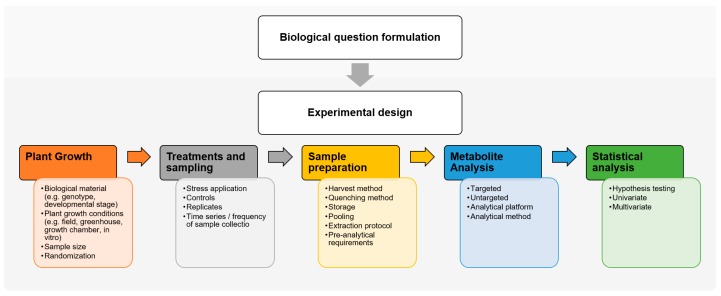
Experimental design and workflow in a plant metabolomics experiment.

The experimental design should ensure that the analytical data derived from the collected biological material would allow answering the initially proposed biological question through a reliable statistical analysis. Therefore, the experimental design (Figure 1) typically includes all variables of the experiment, from the plant growth and treatments (e.g., plant growth conditions, randomization, replicates, controls), sample preparation conditions (e.g., harvested tissue, quenching method, pool material or not, metabolite extraction protocol), and analytical platform (e.g., GC-MS, LC-MS, mass spectrometry imaging, targeted or untargeted approach) to statistical treatments [7,9,34]. Added to these factors, all sources of additional variation (e.g., genotype, sample size, tissue selection, developmental stage, environmental conditions, batch/block effect) should be investigated and minimized to avoid misleading conclusions [7,9,35]. The experimental design should also take into account the time frame of the metabolomics experiment. Because metabolites are highly dynamic (in time and space), a metabolomics study can reflect the steady state (or instant snap-shot) of the metabolism or its dynamic time-course evaluation [9,36,37,38]. In plant metabolomics, due to the destructive nature of the sampling procedure, most conducted studies are transversal (i.e., cross-sectional), where different samples are used for each time point, whereas in human metabolomics, longitudinal studies are fairly common [39,40]. Even if the harvesting procedure is not completely destructive, the wounding effect in plants should be taken into account as it can affect metabolite profiles. Longitudinal studies in plant metabolomics include the analysis of volatile organic compounds (VOCs) through non-destructive headspace techniques (further details in Section 3).

Forest tree experiments are particularly difficult to execute, mainly because of the tree’s long life cycle and lack of genomic tools [41], which in turn leads to highly costly, and time-consuming long-term studies. Thus, a rigorously elaborated experimental design can help to control time and costs and assure that the experiment and respective derived data are reliable and reproducible [42].

#### 2.2.1. Experimental Conditions

The experimental design should clearly define the experimental conditions of the study (i.e., plant growth conditions and treatment(s) to be applied). Plants can be grown under controlled environmental conditions (e.g., growth chambers, nurseries, greenhouses) or in field conditions. From growth chamber to field conditions, there is a gradual decrease in the level of environmental control and a gradual increase in its complexity. Therefore, most metabolomics studies are essentially comparative, i.e., controls (healthy and/or mock treatments in the case of plant–pathogen interactions) vs. treated samples, and always provided that plants are grown under the same conditions [35]. However, in the field, plants are subjected to uncontrolled variations in the environment (e.g., variations in light intensity, temperature, water availability). Despite the plant’s metabolism degree of plasticity acting as a buffer against sudden fluctuations in the environment, this complex set of variables deeply impact the plant’s physiology and metabolism [9], which is often the case of forest tree long-term research. Hence, care must be taken when making comparisons amongst field-grown individuals or even when extrapolating results or establishing correlations between trees grown under controlled and uncontrolled (i.e., field) environmental conditions [43].

In vitro assays are an alternative biotechnological approach to in vivo field studies as it drastically reduces the time needed for the experiment to be conducted and eliminates environmental related fluctuations, allowing the manipulation of single variables in a controlled environment, which is impossible to achieve in field or greenhouse conditions. In vitro cultures have been applied to forest tree research, namely in the establishment of co-cultures to study plant–pathogen interactions, namely co-cultures of *Pinus pinaster* and *Bursaphelenchus xylophilus* as an alternative biotechnological approach to study the pine wilt disease [44] or for rapid clonal propagation of *Populus* spp. [45]. The analysis of plant–pathogen interactions poses a particular challenge in metabolomics studies due to the difficulty in discriminating between plant and pathogen. In this case, in vitro cell co-cultures can be regarded as an alternative dual metabolomics approach to study such metabolite responses in plant–pathogen interactions. This technique allows discrimination between plant cells and pathogens and can further be applied to compare the metabolite response to different pathogenic strains [46]. However, this biotechnological approach should be regarded as a preliminary tool for forest tree research; it is crucial to assess if these systems reflect the real physiological conditions of the plant to only later extrapolate the findings to the whole organism [9]. Another approach to study plant–pathogen interactions, without the need for cell cultures but also allowing the assessment of the spatial distribution of metabolites in plant and pathogen, is with mass spectrometry imaging [47]. However, this technique has not yet been applied in forest tree metabolomics research.

#### 2.2.2. Replicates and Randomization

To compensate for quantitative and qualitative variations in metabolomics analyses, biological replicates are essential for powerful statistical analysis and reliable biological interpretation of the results. Technical replicates can compensate for protocol or instrumental variations but do not improve the statistical analysis of the results [9,48]. In plant metabolomics, the minimum acceptable number of biological replicates should be six [2,49,50]. The biological replicates should be representative of the population under study. For a stronger and significant statistical analysis, the number of replicates needed can be established by power analysis determined from the degree of analytical variance within the populations under study [50]. Statistical power analysis relates sample size, effect size (i.e., the difference of two group means divided by the pooled standard deviation) and significance level to the chance of detecting an effect in a dataset, and thus, should be performed before conducting the experiment as a key step in the experimental design [51,52]. Information for power analysis can be obtained through pilot studies or extrapolated from the literature [51]. Sample size determination modules can be found in bioinformatic tools for metabolite data analysis, such as MetaboAnalyst 3.0, based on the Bioconductor R package Sample Size and Power Analysis (SSPA) and using data from a pilot metabolomic study [52]. However, power analysis is often avoided, and sample size determination becomes driven by sample availability [51]. In the case of limited amounts of sample and high biological variation, pooling samples is a common procedure [7,9,38]. However, this information should be taken into account when performing the data analysis as it may compromise the quality of the data (e.g., a pool containing an odd sample or individual not grown under the same exact conditions).

Randomization is critical for reducing experimental error and biological variability. If working under controlled environmental conditions (e.g., growth chamber), plants should be rotated during the course of the experiment to compensate for variations in light intensity or ventilation that can ultimately affect metabolism and the reproducibility of the data [7,9,53,54]. If plants are grown in a greenhouse or field conditions, variation in environmental conditions is likely to be observed. In all cases, it is crucial to keep a record of all observed changes in the course of the experiment and include them in the metadata and storage databases to ensure data reusability [49,54]. A common strategy to compensate for the impossibility of performing randomization (especially when working with a high number of individuals) is to arrange plants in a block design [9]. In a block design, the individuals are divided into homogeneous groups (i.e., blocks), and treatments are assigned randomly within the block. Treatment comparisons are then performed within blocks because the variability within each block is lower than the variability between blocks. Additionally, harvest should be performed randomly in each block to minimize block effect. The use of an appropriate design is particularly important in forest tree studies because of the long-term nature of the experiments. The variation between blocks (i.e., block factors), such as time, operator, or location, can be later included in the analysis. The randomized complete block design is the standard design pattern because of its simplicity. In this block design, the same number of individuals from each treatment and/or genotype is randomly allocated per block, and act as biological replicates. This block design is most effective when the site is relatively uniform; however, this is rarely the case in forestry studies. To overcome this limitation, other designs, such as the spatially-balanced complete block design [55] or the incomplete block design [56], that allow for better control of heterogeneity are becoming widely popular.

## 3. Sample Preparation for Forest Tree Metabolomics

In metabolomics, as in any analytical science, the sample preparation protocol has a crucial impact on the obtained analytical data. The workflow includes the harvest of the biological material and immediate quenching of metabolism and storing prior sample homogenization and metabolite extraction [2,6,7,34,53,57]. Sample preparation must be meticulously planned to identify potential sources of experimental variation and errors that might compromise data analysis, re-usability of the data, or biological interpretation of the results [6]. To obtain a standard protocol, the sample preparation method should be validated for the plant tissue under study using technical replicate extractions to determine the method precision and quantitative reproducibility [57]. In this section, the importance and challenges of performing harvest and quenching of tree material, especially in field conditions, are discussed, followed by the most common metabolomic workflows in forest tree metabolomics.

### 3.1. Harvest and Quenching

The precise time and process of sampling, or harvest, is a decisive step in a metabolomics experiment because it determines the “metabolic snapshot” of the organism to be analyzed, which directly influences the biological interpretation of the results [6,7,38]. In addition, the harvest should be performed as quickly as possible to avoid diurnal variations and the loss of metabolites with high turnover rates [2,6,7,37,50]. When working with forest tree species, samples are, in most cases, collected in the field and should be properly stored until lab processing. Ideally, biological samples should be immediately frozen in liquid nitrogen to avoid loss or degradation of biomolecules. However, this method is practically impossible to apply to samples harvested in natural ecosystems. In these cases, the best approach is to use silica gel to dehydrate the samples, thus stopping biochemical reactions [58]. Nevertheless, volatile compounds are often difficult to recover. In addition to the sample storage under field conditions, data relative to the exact geographical location and edaphic–climatic conditions should be described in as much detail as possible, to provide a more complete characterization of the provenance [58].

After the harvest, the second step in sample preparation is to instantly quench metabolism, usually by flash-freeze, using liquid nitrogen (shock freezing). Quenching is a crucial step in metabolomics workflows to immediately stop the metabolism and avoid further changes occurring in the sample, such as metabolite degradation or variations in their concentration, chemical, or physical properties [2,6,34]. Other methods include freeze-drying or the use of ice-cold methanol. Despite the risk of lower extraction reproducibility when working with frozen fresh samples, freeze-drying is a slower process that can lead to the production of artifacts, and potentially lead to the irreversible adsorption of metabolites on cell walls and membranes [36,59]. Freeze-drying can be a convenient method when weighing large sample sets, but it has been reported to reduce extraction yields by 25% [60]. In a comparative phytohormone quantification study between fresh-frozen and freeze-dried plant material, namely needles of *P. pinaster*, leaves of *Eucalyptus globulus*, and cotyledons of *P. pinea*, higher recoveries were obtained when using fresh material [61]. Freeze drying methods enhance lipid extraction by eliminating all the water, and consequently, generating a more complex matrix making phytohormone quantification in the presence of these interferences difficult. The appropriate sample treatment should always be evaluated according to the plant tissue under study.

In plant metabolomics, quenching is usually followed by homogenization of the sample, typically using a pestle and mortar or ball mill for plant–cell–wall breakage and sample weighing. These steps are always performed under liquid nitrogen to prevent tissue from thawing [2,6,7].

### 3.2. Metabolite Extraction

The choice of a metabolite extraction protocol is extremely important in metabolomics studies as it can directly affect the metabolite coverage and metabolite concentration. Ideally, a metabolite extraction protocol aims to (i) efficiently isolate metabolites from the sample in a high-throughput manner; (ii) be as non-selective as possible to ensure adequate metabolite coverage; (iii) prevent metabolite loss or degradation; (iv) be reproducible; (v) remove interferents that can affect the analysis; (vi) be compatible with the chosen analytical technique; and, when necessary, (vii) concentrate low abundance metabolites before analysis [2,34,59,62]. Typically, a metabolomics experiment follows two alternative approaches, targeted or untargeted. In targeted metabolomics, a well-defined group of known annotated metabolites is identified, whereas an untargeted approach aims to provide an overview of all the measurable analytes in the biological sample, including unknown compounds. However, it is important to highlight that, due to the vast variety of metabolites present in the plant metabolome, at different concentration levels and with distinct physical–chemical properties, it is impossible to extract the whole range of metabolites using a single extraction protocol [2,6,38].

In a metabolomics extraction protocol, several aspects have to be considered, namely the choice of an appropriate solvent system, solvent solubility, solvent to sample ratio, duration, and temperature of the extraction [2,5,6,36,63]. The choice of the appropriate solvent depends not only on the metabolite properties to be extracted but also has to meet the specific requirements of the analytical platform to be used (e.g., GC-MS, LC-MS). The exception is the use of headspace extraction (e.g., solid-phase microextraction, SPME) for the extraction of volatile components without the need for solvents [5,38]. For LC-MS, the only main limitation is that the solvent in which the sample is injected must be miscible and should be similar to the LC mobile phases used. For the typical reverse-phase separations, solvents used are generally aqueous eluents with 5–50% of an organic solvent (e.g., methanol, acetonitrile) [6]. Moreover, the addition of stable isotopically labeled internal standards to the extraction buffer (e.g., ^15^N and ^13^C labeling strategies) in targeted plant metabolomics approach is an excellent tool (i) to monitor extraction reproducibility; (ii) to compensate for ionization suppression/enhancement effects, accuracy, precision, and matrix effects of an analytical method or during method validation; and (iii) for normalization in data analysis [9,62,64].

#### 3.2.1. GC-MS Metabolite Profiling

In forest tree research, GC coupled to either a time-of-flight MS (GC-TOF-MS) or a fast scanning quadrupole MS (GC-qMS) have often been employed for high-throughput plant primary metabolite profiling allowing the measurement of complex mixtures of primary metabolites (e.g., organic acids, sugars, sugar alcohols, amino acids) in a single extract [8]. GC-TOF-MS shows numerous advantages over GC-qMS, namely, higher mass accuracy, higher duty cycles, and faster acquisition rates that ultimately contribute to a better deconvolution of overlapping peaks and higher sample throughput [2,3,65].

Despite the low number of publications in forest tree metabolomics, when compared to other omics studies [66], GC-TOF-MS has been the method of choice for the primary metabolite profiling of forest tree responses to abiotic and biotic stresses [24,25,58,67,68,69] as well as other plant growth-related processes [17,26,70,71,72,73,74,75,76,77]. In these forest tree metabolomics studies, as for plant metabolomics in general, primary metabolites for GC-TOF-MS analysis are commonly extracted using the well-established chloroform:methanol:water extraction protocol, with minor optimization variations across studies (e.g., time of extraction, temperature, solvent ratio, or addition order), and further derivatized with *N*-methyl-*N*-(trimethylsilyl)trifloracetamide (MSTFA), containing a mixture of fatty acid methyl esters (FAMEs) with different chain length as time standards (i.e., standard for retention time calibration) [2,34,50,63]. This two-phase solvent system has the advantage of fractionating the metabolites from a single sample into a polar aqueous phase (methanol:water) and a lipophilic organic phase (chloroform), which can be further analyzed separately [63].

Additional GC-qMS studies in forest tree species include the profile of the volatile fraction, namely volatile organic compounds (VOCs) and essential oil (EO). This volatile fraction is mainly dominated by terpenoids, phenylpropanoids/benzenoids, fatty acid derivatives, and amino acid derivatives [78]. Despite the similarity in their qualitative chemical composition, the relative amounts of metabolites found in these two volatile fractions can differ greatly due to the distinct extraction processes involved [79]. VOCs are commonly collected with headspace techniques (e.g., SPME), while EOs are obtained exclusively with hydro-, steam- or dry-distillation, or in the case of citrus fruits, mechanically without heating [80]. EO screening studies are popular among forest tree species, namely *Eucalyptus* and *Pinus* spp., mainly due to the occurrence of EO chemotypes [81,82,83,84]. The non-destructive nature of headspace techniques, such as SPME, allow for time-course evaluation of VOCs emission and have been widely applied in forest tree research not only for plant chemotype classification but also for plant–pathogen interactions or plant–insect communication [85,86]. This technique simply requires the optimization of the type of fiber, exposure time and temperature, and desorption time and temperature [87].

#### 3.2.2. LC-MS Metabolite Profiling

In forest tree research, LC-MS instruments have also been used for untargeted secondary metabolite profiling and phytohormone quantification studies. The focus of these studies was related with abiotic stress responses [19,20,21,22,23,24,67,88,89]; and to a smaller extent to biotic stress responses [90,91] and plant growth and developmental processes [77,92,93].

Metabolite extraction for LC-MS untargeted analysis are usually performed using a simple protocol based on methanol [19] or methanol:water 80:20, *v*/*v* [20,91] or 50:50, *v*/*v* [90] as extraction solvents. However, most untargeted secondary metabolite profiling studies in forest tree metabolomics research are performed in combination with GC-qMS or GC-TOF-MS primary metabolite profiling, ultimately allowing for more comprehensive coverage of the tree metabolome. In these cases, the chloroform:methanol:water two-phase solvent system is used, and the polar phase is evaporated to dryness and used for both GC-MS (after derivatization) and LC-MS metabolite profiling (after reconstitution in methanol:water) [21,77,88,89]. To take full advantage of the chloroform:methanol:water two-phase solvent system, the non-polar metabolites (lipophilic fraction) are analyzed by GC-MS after derivatization, for example, with tertmethyl–butyl–ether (MTBE) and trimethylsulfoniumhydroxide (TMSH) [23,92].

LC coupled to triple quadrupole-MS (LC-QqQ-MS) has been often employed in method development to quantify phytohormones in plant tissues [94]. Delatorre and co-workers [61] developed and validated an LC-QqQ-MS analytical method to quantify 20 phytohormones in forest tree species tissues, using 2-propanal:water:hydrochloric acid (2:1:0.002 *v*/*v*/*v*) and dichloromethane for a two-phase solvent system, a protocol originally described by Pan and co-workers [95]. Other metabolite extraction protocols for phytohormone quantification in forest tree species include a modified version of the well-established solvent extraction protocol described by Bieleski [96], namely methanol:water:formic acid (15:4:1, *v*/*v*/*v*) [97], methanol:water (80:20, *v*/*v*) [98,99], methanol:water:acetic acid (90:9:1, *v*/*v*/*v*) [16], or water:diethyl ether:acetic acid [22]. The Bieleski solvent extraction protocol [96] has been used for the extraction of phytohormones, particularly cytokines [100].

### 3.3. Pre-Analytical Requirements

In targeted and untargeted metabolomic approaches, the presence of matrix interferents can hinder the MS-based metabolite analysis by adding further complexity in regards to metabolite ionization and interfering molecules that influence the signal response of the metabolites under study. Although in targeted approaches, the use of stable isotopically labeled internal standards can compensate for matrix-induced ionization effects, the availability of standards in untargeted approaches can be very limited. In both cases, metabolite concentration and further sample clean-up steps to remove interferents might be necessary. Solid-phase extraction (SPE) is the method of choice in these cases [5]. SPE is a simple sample preparation technique based on the removal of analytes from a liquid sample by retention on a solid sorbent (e.g., silica, alkylated silica), based on the functional group interactions of the analytes, flowing solvent, and the solid sorbent [5,6,101]. The retained analytes are subsequently eluted from the sorbent using a solvent or solvent mixture with sufficient elution strength. Because it involves the use of large amounts of solvents and requires several steps of concentration, this technique is time-consuming and not high-throughput, resulting in an added risk of losing components during the process. To improve sample high-throughput and reproducibility, 96-well solid-phase extraction plates, and robotic SPE-MS systems have been developed and are commercially available [101]. An SPE step is often included in sample preparation for MS-based phytohormone analysis in forest tree studies to remove interfering components from the matrix, such as pigments, resinic acids, terpenes, carotenoids, flavonoids, cellulose, and lipids, and increase the recovery rates of the phytohormones under study [61,64,99].

## 4. The Importance of Forest Tree Metadata Standardization

Advances in high-throughput MS-based platforms have been responsible for the generation of extremely large metabolomics datasets. For a comprehensive understanding of biochemical pathways and regulatory networks involved in different plant processes and responses, metabolomics datasets can be further integrated with other “omics” studies (e.g., transcriptomics, proteomics), providing the data is available in a standardized and reproducible way. Thus, the description of metabolomics studies should include all the information needed to allow the repetition of the experiment and the re-usability of the data.

To promote standardization of all stages of a metabolomics analysis (i.e., experimental design, biological context, chemical analysis, and data processing) and ensure metadata consistency, in 2007, members of the metabolomics community established the Metabolomics Standard Initiative (MSI) [102,103]. The MSI aimed at reporting standards and provide a clear description of the biological system under study and of the metabolomics analysis workflows to allow data to be efficiently applied, shared, and reused. A decade later, a set of guideline principles known as the FAIR principles (i.e., Findable, Accessible, Interoperable, and Re-usable) were also designed to assure good (meta)data management by data holders and data publishers [104]. ELIXIR, the European infrastructure for biological data (https://elixir-europe.org), has also brought together several communities (e.g., plant sciences, metabolomics) with the common interest of dealing with the increasing complexity of data, ultimately making data easier to find, analyze and share. Challenges currently faced by the metabolomics community, namely (i) minimum information standards and early data capture; (ii) global spectral databases; (iii) tools and standards registries; (iv) compound identifier mapping; (v) omics data integration, and (vi) metabolite identification, have also been reported by ELIXIR in a dedicated workshop [105]. However, despite these initiatives, the compliance with these reporting standards still varies greatly across public repositories [106], and data and metadata sharing remain a critical issue in metabolomics publications [107,108].

Within the field of plant metabolomics, forest tree metabolomics studies present additional challenges concerning the standardization of metadata. Forest trees are species with long life cycles, and details of the experimental metadata (e.g., parental original or field growth conditions) are often not described. To re-use data derived from these studies, the description of the metadata should include detailed information of the harvested material (e.g., geographical location, growth conditions, biological growth stages, and phenological parameters) [8]. These parameters might reflect adaptive traits mediated by epigenetic changes that affect the material under study [109,110]. Thus, as epigenetic changes affect the transcriptome, proteome, and ultimately the metabolome, the integration of these “omics” data strongly depends on the availability of detailed information of the harvested material. Plant phenotyping has been developed significantly over the past years due to the progress in novel sensors, automation tools, and quantitative data analysis methods. Yet, the consequent increase of data generation is still a struggle for the standardization of data acquisition and its re-usability [111].

The urge for metadata standardization for plant phenotyping experiments has been addressed by community-driven projects, for example, the MIAPPE project (Minimal Information About a Phenotypic Experiment), the ISA framework (investigation, study, assay) [112,113] or the GnpIS data repository (genetic and genomic information system) [114]. MIAPPE is available as a checklist of metadata to adequately describe a plant phenotyping experiment and as software to validate, store, and disseminate MIAPPE-compliant data (https://www.miappe.org/). In early 2019, MIAPPE version 1.1 was released as an extended version to include woody plants and compatibility with other phenotyping frameworks (e.g., ISA framework), which represents an important step towards the standardization of forest tree metadata. By providing curated databases, and in accordance with the FAIR principles, these platforms allow data and metadata to be easier to find, integrate, and analyze. Due to the amount of data generated in forest tree studies, dedicated databases or extensions to existing databases have been developed. Dedicated tree databases (e.g., PlantGenIE, TreeGenes, and Hardwood Genomics Project) covering mostly genetic data have now the goal of associating phenotypic and environmental data [115].

Integrated into crop ontology, the woody plant ontology has been established as an additional platform for the annotation of forest tree metadata [116]. The woody plant ontology adds a set of definitions to the existing crop ontology to describe specific tree traits (e.g., secondary growth in wood and cork formation). Studies with forest tree species, particularly in field experiments, can take several years to develop, and it is crucial to adequately annotate all metadata across the tree’s long life cycles. Such case studies that use machine-accessible metadata can be found in the literature. One example is the forest growth measurements from individual *Picea abies* trees over the course of 109 years [117]. The metadata file describing the reported data is openly available in an ISA-tab format and can be further used to analyze and validate forest growth.

## 5. Conclusion

Metabolomics studies are often regarded as the ultimate response of biological systems to genetic or environmental alterations. Although most MS-based plant metabolomics research is performed on crop and non-tree model species, in recent years, studies on forest tree species have generated particular interest, especially after major genomics breakthroughs in forest tree research (e.g., availability of the *Populus trichocarpa* reference genome in 2006). In this area, MS-based metabolomics represents a unique opportunity to explore the forest tree’s adaptation to environmental fluctuations as well as other economic and ecological relevant developmental processes. However, and as previously discussed, to successfully obtain significant data from metabolomics analyses, it is crucial to have a well-planned experimental design and an appropriate sample preparation. Any metabolomics study should include, in great detail, a clear description of the design of the experiment as well as of other technical parameters. Despite the struggles, continuous efforts from the metabolomics scientific community have been made to ensure data and metadata reproducibility between laboratories and to promote the availability of curated databases and repositories containing high-quality data (including dedicated woody species platforms).
